# The aberrant expression of rhythm genes affects the genome instability and regulates the cancer immunity in pan‐cancer

**DOI:** 10.1002/cam4.2834

**Published:** 2020-01-12

**Authors:** Jian Zhou, Xinhui Li, Minghui Zhang, Ji’nan Gong, Qi Li, Baocong Shan, Tianzhen Wang, Lei Zhang, Tongsen Zheng, Xiaobo Li

**Affiliations:** ^1^ Department of Pathology Harbin Medical University Harbin China; ^2^ College of Bioinformatics Science and Technology Harbin Medical University Harbin China; ^3^ Department of Oncology Chifeng City Hospital Chifeng China; ^4^ Department of Gastrointestinal Medical Oncology Harbin Medical University Cancer Hospital Harbin China

**Keywords:** cancer immunity, circadian rhythm genes, genome instability, microsatellite instability

## Abstract

Although emerging studies showed that certain rhythm genes regulate cancer progression, the expression and roles of the vast majority of rhythm genes in human cancer are largely unknown, and the hallmarks of cancer regulated by rhythm genes have not been detected. In this study, we detected the expression changes of rhythm genes in pan‐cancer and found that almost all rhythm genes mutated in all cancer types, and their expression level was significantly altered partially due to abnormal methylation, and several rhythm genes regulate the expression of other rhythm genes in various cancer types. Furthermore, we revealed that rhythm genes are significantly enriched in genome instability and the expression of certain rhythm genes is correlated with the tumor mutation burden, microsatellite instability, and the expression of DNA damage repair genes in most of the detected cancer types. Moreover, rhythm genes are associated with the infiltration of immune cells and the efficiency of immune blockade therapy. This study provides a comprehensive understanding of the roles of rhythm genes in cancer immunity, which may provide a novel method for the diagnosis and treatment of cancer.

## Graphical abstract

We reported that almost all rhythm genes mutated in all cancer types, and their expression level was significantly altered partially due to abnormal methylation and a series of functions of rhythm genes in tumor development. Rhythm genes are associated with the infiltration of immune cells and the efficiency of immune blockade therapy. This study provides a comprehensive understanding of the roles of rhythm genes in cancer immunity, which may provide a novel method for the diagnosis and treatment of cancer.

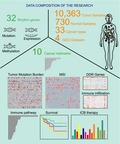

## 1 INTRODUCTION

The vast majority of the creatures on earth is active during the day and repose at night due to the biological clock of about 24 hours, which is the circadian rhythm driven by the regular oscillation of the expression of set genes. Detection of the molecular mechanisms regulating circadian rhythm led to the identification of rhythm genes, which had been granted the Nobel Prize in 2017. The rhythm genes not only control the circadian rhythm but also regulate the physiology and behavior of the organism, such as the sleeping model, hormone release, feeding behavior, body temperature and blood pressure.^1^, ^2^ Recent studies have shown that aberrant circadian rhythm is associated with various human diseases including cancer.^3^

Currently, the roles of rhythm genes in regulating cancer progression are emerging. Firstly, it has been revealed that destroyed circadian rhythm results in an increased incidence of certain malignant tumors, such as breast carcinoma, Lung cancer.^4^, ^5^ Secondly, the aberrant expression of rhythm genes has been frequently reported in many human cancer types. For instance, the expression of Bmal1 is elevated in hepatoma^6^; altered expression of PER2 due to abnormal methylation is observed in glioma^7^; mutations of Ck1δ and Ck1ε are associated with an increased incidence of many cancer types.^8^, ^9^, ^10^, ^11^, ^12^, ^13^, ^14^Thirdly, it has been demonstrated that the expression change in certain rhythm genes regulates cancer progression. For example, the aberrant expression of Dec1, Dec2, and PER1 plays an important role in the migration and proliferation of malignant epithelial cells^15^; destroyed normal expression of PER2 is beneficial to malignant transformation of glioma cells.^7^ Finally, in the mechanism, it has been revealed that rhythm genes can regulate the cell cycle, apoptosis, DNA damage repair and cancer‐associated inflammation during cancer progression.^16^, ^17^, ^18^ Although the association between cancer progression and aberrant expression of certain rhythm genes has been well studied, the expression and roles of the vast majority of rhythm genes in various cancer types are largely unknown. Moreover, the major hallmarks of cancer regulated by rhythm genes have not been intensively detected previously.

In this study, we analyzed the expressional alteration, mutation, and methylation status of rhythm genes in pan‐cancer through a series of statistical and bioinformatics methods. We also analyzed the association between the expression of rhythm genes and hallmarks of cancer and prognosis of pan‐cancer, respectively. Particularly, we investigated the relationship between the expression of rhythm genes and the level of immune infiltration, and further detected their effects on immune blockade therapy in some cancer types based on published datasets.

## 2 MATERIALS AND METHODS

### 2.1 Collection of the expressional data of rhythm genes in pan‐cancer

We preliminary obtained 184 rhythm relative genes by searching for gene function annotation file download from NCBI (ftp://ftp.ncbi.nlm.nih.gov/), and finally confirmed 32 rhythm genes (Table S1) via manually checking the gene database (https://www.ncbi.nlm.nih.gov/gene/) and related literature (https://www.ncbi.nlm.nih.gov/pubmed/). The expression of these rhythm genes in pan‐cancer was collected from The Cancer Genome Atlas database (TCGA: https://www.cancer.gov/),which contains 10,363 cancer samples and 730 normal samples from 33 cancer types (Table S2).

### 2.2 Analyzing the expressional alteration and the status of methylation and mutation of rhythm genes in pan‐cancer

We used the *t* test to analyze the differential expression of rhythm genes between the cancer samples and normal samples in 14 cancer types. The methylation status of rhythm genes in different cancer types was obtained from MethHC database.^19^ Pearson correlation analysis was employed to investigate the correlation between the expression and methylation level of rhythm genes in 13 cancer types with both expression and methylation data. Mutation data of rhythm genes from 31 cancer types were collected from the cBioPortal database (http://www.cbioportal.org/).We analyzed the mutation of rhythm genes with R package *ggplot2* by R3.6.1.

### 2.3 Analyzing the transcriptional regulation of rhythm genes

To assess the interactional regulation among circadian rhythm genes, we identified 12 transcription factors in the rhythm genes and then detected the transcriptional regulation of them on all rhythm genes by using a CHIP‐seq database Cistrome DB (http://cistrome.org/db/#/)^20^, ^21^ and the regulatory relationships are presented in a network form drawing by Cytoscape 3.6.1.^22^

### 2.4 Tumor Mutation Burden (TMB) and Microsatellite instability (MSI) data collection

We download the Tumor somatic mutation data from TCGA, the correlation between the expression of rhythm genes and the level of TMB is analyzed by Pearson correlation. DNA recombination repair genes were acquired from a previous study,^23^ and their expression in pan‐cancer were downloaded from TCGA, and the expressional correlation between the rhythm genes and recombination repair genes is analyzed by Pearson correlation. The MANTIS tool was used to assess the microsatellite instability of the tumor samples.^24^, ^25^ Samples of each cancer type were divided into the MSI‐Low and MSI‐High group, respectively, with 0.4 as the threshold,^25^ and the *t *test was used to compare the expression level of rhythm genes in the MSI high and low groups.

### 2.5 Investigating the hallmarks of cancer regulated by rhythm genes in pan‐cancer

In order to investigate the major hallmarks of cancer regulated by rhythm genes in pan‐cancer, we analyzed the enrichment relationship between the expression of rhythm gene sets and 10 different gene sets indicating individual hallmark of cancer by using hypergeometric test.^26^ Seventeen immune‐related pathways were downloaded from the ImmPort Private Data website (https://immport.niaid.nih.gov/home), and the relationship between rhythmic genes and these immune pathways was also analyzed using hypergeometric test.^27^

### 2.6 Analyzing the correlation between the expression of rhythm genes and the infiltration of immune cells

The mRNA expression profile data of 33 cancer types were downloaded from the TCGA database which was used for predicting the infiltration of immune cells in different cancer types. All the data processing is performed by the *R* 3.6.1. Totally 547 signature genes indicating 22 immune cell subtypes were used to evaluate the degree of immune cell infiltration. And the detailed methods and analysis tool have been reported previously.^28^, ^29^ The correlation and significant *P*‐value of rhythm genes with tumor cell immune infiltration were calculated by the Pearson correlation coefficient. All graphs were made by R package *ggplot2*.

### 2.7 Survival analysis

We analyzed the impact of the rhythm genes on the survival of cancer patients in 33 cancer types. Survival analysis was performed by using Cox proportional hazards regression model. Based on the expression of each rhythm gene, cancer samples were divided into high and low groups with a threshold of 50%, and then the Cox risk regression was analyzed by using the Log‐rank test. The Cox proportional hazard ratio and the 95% confidence interval information also have been included in the survival map. The thresholds for high/low expression level cohorts can be adjusted.^30^

### 2.8 Analyzing the impact of rhythm genes on the ICB therapy in pan‐cancer

Data of immunological checkpoint blockade (ICB) treatment with anti‐PD‐1 was obtained from the http://www.ncbi.nlm.nih.gov/geo/query/acc.cgi?acc=GSE78220 dataset,^31^ which contains gene expression profiles and treatment information for 28 melanoma patients who received therapy with Pembrolizumab, and another dataset http://www.ncbi.nlm.nih.gov/geo/query/acc.cgi?acc=GSE79691,^32^ which contains gene expression profiles and treatment information for 10 metastatic melanoma patients treatment with Nivolumab. We divided the samples from http://www.ncbi.nlm.nih.gov/geo/query/acc.cgi?acc=GSE78220 and http://www.ncbi.nlm.nih.gov/geo/query/acc.cgi?acc=GSE79691 into two groups, respectively, according to the response to treatment with anti‐PD‐1, and then analyzed the relationship between the expression of rhythm genes and the effect of anti‐PD‐1 immunotherapy by using the *t *test.

The anti‐CTLA4 therapy data were obtained from http://www.ncbi.nlm.nih.gov/geo/query/acc.cgi?acc=GSE91061,^33^ which contains 109 RNASeq samples (58 post‐treatment and 51 pretreatment) from 65 melanoma patients. We analyzed the expression change of rhythm genes after anti‐CTLA4 treatment by using the *t* test*.*

http://www.ncbi.nlm.nih.gov/geo/query/acc.cgi?acc=GSE117358 dataset containing 24 mesothelioma samples and 24 kidney cancer samples of mouse that were separately treated with PD‐1 and CTLA4 antagonists was employed to detect the expression change of the rhythmic genes between the response group and the nonresponse group by using the Wilcoxon rank‐sum test.

## 3 RESULTS

### 3.1 Rhythm genes are highly mutated in pan‐cancer

**Figure 1:**
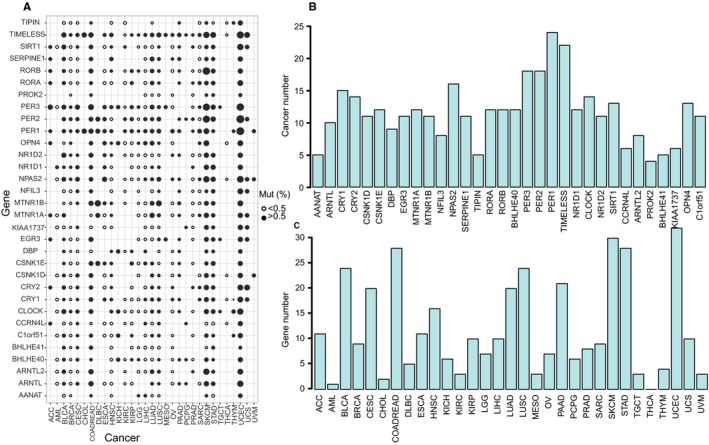
High mutation rate of rhythm genes is detected in pan‐cancer. A, Mutation status of the rhythm genes in pan‐cancer. The size of the dots indicates the magnitude of the mutation rate. B, The mutation frequency of each rhythm gene in pan‐cancer reflected by the number of cancer types with a mutation rate >0.5%. C, The mutation frequency of all rhythm genes in each cancer type reflected by the number of rhythm genes with a mutation rate >0.5%

Studies have shown that mutations in some rhythm genes increased the risk of cancer,^8^, ^9^, ^10^, ^11^, ^12^, ^13^, ^14^, ^34^, ^35^ thus we detected the mutation rate of rhythm genes in pan‐cancer. We found that most of the rhythm genes show a significant high mutation rate among all the cancer types tested, with a mutation rate more than 0.5% as a criterion for significant mutation,^36^ we revealed that most of the rhythm genes are significantly mutated in almost all cancer types (Figure 1A). Among these rhythm genes, PER1, PER2, PER3, and TIMELESS are the most frequently mutated genes in pan‐cancer (Figure 1B). And more than 70% of rhythm genes are significantly mutated simultaneously in nine cancer types (Figure 1C). Especially, all the rhythm genes are mutated in UCEC with a mutation rate generally higher than 2.5% (Figure 1A). These results suggested that rhythm genes are highly mutated in pan‐cancer, which may contribute to the tumorigenesis of various cancer types.

### 3.2 The expression of most rhythm genes are significantly changed partially due to abnormal methylation in pan‐cancer

**Figure 2:**
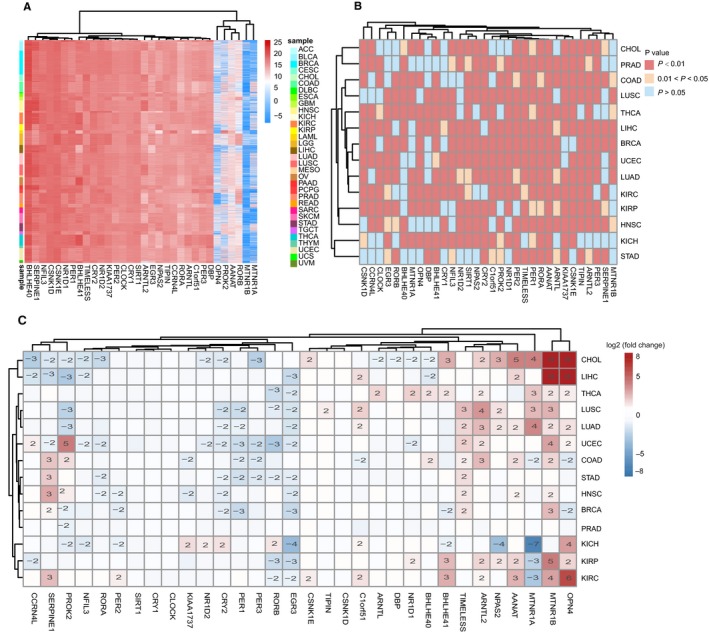
The expression of most rhythm genes are significantly changed in pan‐cancer. A, The expression pattern of rhythm genes in 10 363 cancer samples from 33 cancer types is detected by using clustered analysis and is largely clustered into four groups. B, Comparing the expression of rhythm genes in normal and tumor samples. The *P*‐value of the *t* test is performed by −log10 conversion. The red and orange colors indicate the statistical significance. C, Expressional fold change of rhythm genes in cancer tissues compared with normal samples. The expressional value of each sample is performed by log2 conversion

We employed the clustered analysis to detect the expressional alteration of rhythm genes according to the expression values of these rhythm genes in 10 363 cancer samples from 33 cancer types obtained from TCGA. The results showed that the expressional pattern of the rhythm genes in pan‐cancer could be largely clustered into four groups, and three groups are relatively close, whereas another group of rhythm genes shows a heterogenous expressional pattern (Figure 2A). Furthermore, we detected the expression differences of 32 rhythm genes in cancer samples and normal samples from 14 cancer types, and the results showed that the expression of most of the detected rhythm genes are significantly changed (Figure 2B), among them, OPN4, MTNR1B, MTNR1A, AANTN, NPAS2, ARNTL2, and TIMELESS are significantly upregulated, whereas EGR3, RORB, PER3, PER1, and CRY2 are significantly downregulated in most cancer types (Figure 2C).

**Figure 3:**
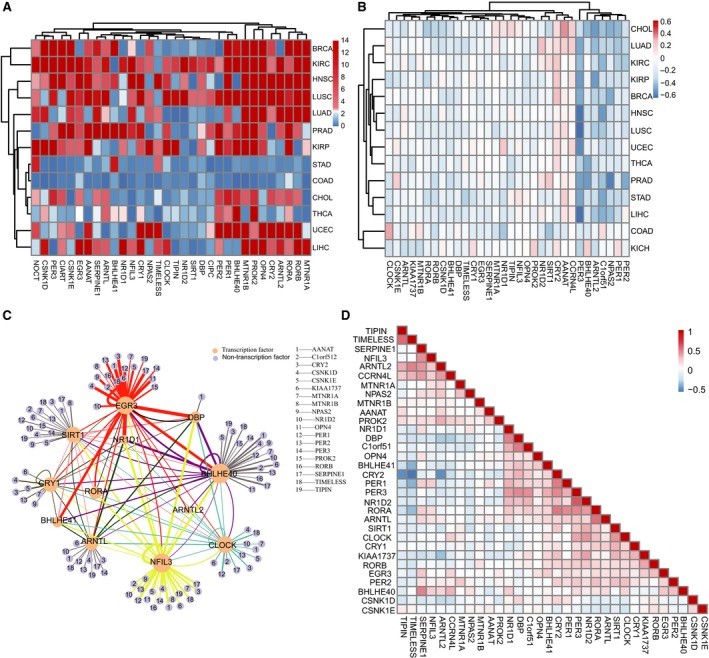
The expression change of rhythm genes partially due to abnormal methylation in pan‐cancer. A, Comparison of the methylation level of rhythm genes between cancer and normal samples with *t* test. *P*‐value is performed ‐log10 conversion. B, Correlation analysis between expression and methylation of rhythm genes in different cancer type with Pearson correlation. C, The regulatory network of rhythm genes functioning as transcription factors. The size of the circle indicates the number of rhythm genes regulated by this transcription factor. D, Expressional correlation among rhythm genes in pan‐cancer analyzed with Spearman correlation

Since the abnormal methylation of the gene promoter contributes to the expression change of genes in cancer samples, we analyzed the promoter methylation of rhythm genes in different cancer types. We found the abnormal methylation of the rhythm genes presents in multiple cancer types, especially in BRCA, KIRC, HNSC, LUSC, LUAD, PRAD, KIRP, UCEC, and LIHC (Figure 3A). Moreover, we used Pearson correlation to analyze the correlation between the expression and methylation level of rhythm genes in 13 cancer types, and revealed that the methylation level of PER1, PER2, PER3, NPAS2, BHLHE40, C1orf51, and ARNTL2 are significantly negatively correlated with their expression level, respectively (Figure 3B), which may partially explain the expression changes of these rhythm genes in cancer samples.

### 3.3 Rhythm genes regulate the transcription of themselves in pan‐cancer

In addition to investigating the roles of methylation in the expression of rhythm genes in pan‐cancer, we also detected the expression change of rhythm genes at the transcriptional level. We identified 12 of the rhythm genes function as a transcription factor, and then we investigated if each of them regulating the transcription of all rhythm genes. We found that most of these transcription factors regulate the transcription of other rhythm genes. For example, BHLHE40, NFIL3, and EGR3 regulate the transcription of more than 90% rhythm genes, respectively. Moreover, several of them, including BHLHE40, NFIL3, EGR3, CLOCK, and CRY1, also regulate the transcription of themselves (Figure 3C). Furthermore, by using Pearson correlation analysis, we confirmed that most of the rhythm genes are positively correlated with the expression of other rhythm genes in pan‐cancer (Figure 3D). These results suggested that the rhythm genes may regulate the progression of cancer cells integratively instead of separately.

### 3.4 Rhythm genes are associated with genome instability and infiltration of immune cells in pan‐cancer

**Figure 4:**
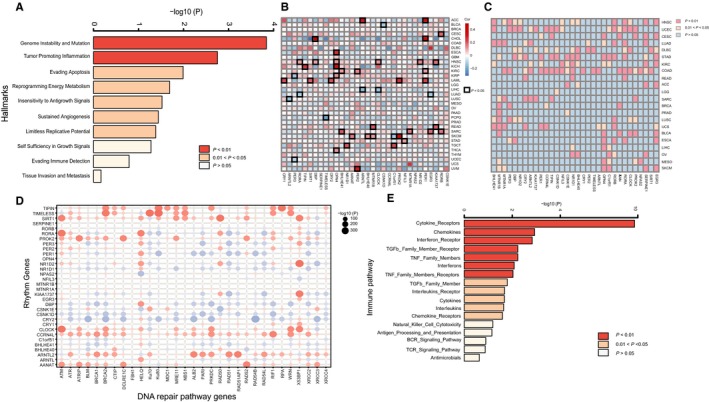
Rhythm genes are associated with genome instability of cancer cells. A, Enrichment analysis of rhythm genes with ten hallmarks of cancer shows that rhythm genes are associated with genome instability and mutation. Different color indicates a different *P*‐value. B, Correlation analysis between rhythm genes and tumor mutant burden (TMB) in pan‐cancer. Red means a positive correlation, blue means a negative correlation, and the border‐box indicates statistic significance. C, Analyzing the relationship between rhythm genes and microsatellite instability (MSI) by comparing the expression level of rhythm genes in high MSI and low MSI group in pan‐cancer. Different color indicates a different *P*‐value. D, Correlation analysis between the expression of rhythm genes and DNA recombination repair genes. Red means a positive correlation, blue means a negative correlation, and the size of dots represents the magnitude of the *P*‐value. E, Enrichment analysis of rhythm genes with immune pathways. *P*‐value is performed −log10 conversion

To evaluate the powerful roles of rhythm genes in cancer progression, we analyzed the relationship between rhythm genes and the hallmarks of cancer. The results showed that the rhythm genes are significantly enriched in three hallmarks of cancer, including genome instability and mutation, tumor‐promoting inflammation, and evading apoptosis (Figure 4A).

To confirm the roles of rhythm genes in regulating genome instability and mutation, we detected the correlation between the expression of rhythm genes and the level of tumor mutation burden (TMB) in pan‐cancer. We found that the expression of certain rhythm genes is positively correlated with the TMB in most of the detected cancer types, especially in HNSC, KIRC, LAML, SARC, and SKCM, whereas the expression of some rhythm genes is negatively correlated with the TMB in certain cancer types (Figure 4B). Consistently, we also revealed that the expression level of certain rhythm genes is significantly different in the high MSI and low MSI group. There are six cancer types (HNSC, UCEC, DLBC, STAD, KIRC, and COAD) that show more than 1/3 rhythm genes differently expressed in the high and low MSI group. Whereas there are nine rhythm genes (MTNR1B, NR1D1, RORB, RORA, OPN4, C1orf51, PROK2, SERPINE, and EGR3) that are differently expressed in the high and low MSI group in more than 1/3 cancer types (Figure 4C). Moreover, we analyzed the expressional correlation of rhythm genes and the DNA recombination repair pathway genes. We revealed that the expression of TIPIN, TIMELESS, SIRT1, PROK2, CLOCK, CCRN4L, ARNTL2, and AANAT are significantly positively correlated with the level of DNA recombination repair genes, whereas the expression of PER3, PER1, NR1D1, DBP, CSNK1D, CRY2, C1orf51, and BHLHE41 are significantly negatively correlated with the level of DNA damage repair genes (Figure 4D). These results suggest that rhythm genes play an important role in DNA damage repair and genome instability in pan‐cancer.

**Figure 5:**
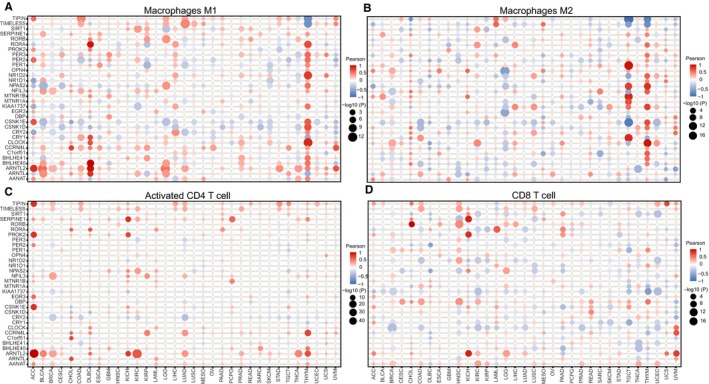
Rhythm genes are associated with immune cell infiltration in pan‐cancer. The correlation between the expression of the rhythm genes and the infiltration level of type 1 macrophages (M1, Figure 5A), type 2 macrophages (M2, Figure 5B), CD4^+^ T cells (Figure 5C), and CD8^+^ T cells (Figure 5D).The size of the nodes represent the magnitude of significance, red means a positive correlation, and blue means a negative correlation

Since genome instability and mutation increases the production of tumor‐associated antigens, thus the expression of rhythm genes may be associated with cancer immunity in the cancer microenvironment. To test this hypothesis, we analyzed the relationship between rhythm genes and immune pathways, and the correlation between rhythm genes and four tumor‐infiltrating lymphocytes (TIL), including M1 macrophages, M2 macrophages, CD4^+^ T cells and CD8^+^ T cells, respectively. We found those rhythm genes are significantly enriched in several immunity pathways, including interferon signaling pathway, TGF‐β signaling pathway, and TNFα signaling pathway et al (Figure 4E). Additionally, we showed that almost all rhythm genes are correlated with the infiltration of all tested immune cells in pan‐cancer (Figure 5A‐D**)**. Among them, the expression of TIPIN, TIMELESS, ARNTL2, ARNTL, and AANAT are positively correlated with the infiltration level of tumor‐infiltrating lymphocytes in pan‐cancer, respectively. On the contrary, the expression of PER2, PER3, CRY1, KIAA1737, and BHLHE41 are negatively correlated with the infiltration level of four tumor‐infiltrating lymphocytes in pan‐cancer. These results suggest that rhythm genes are involved in the regulation of cancer immunity and cancer‐associated inflammation.

### 3.5 The expression of rhythm genes predicts the prognosis of cancer patients

**Figure 6:**
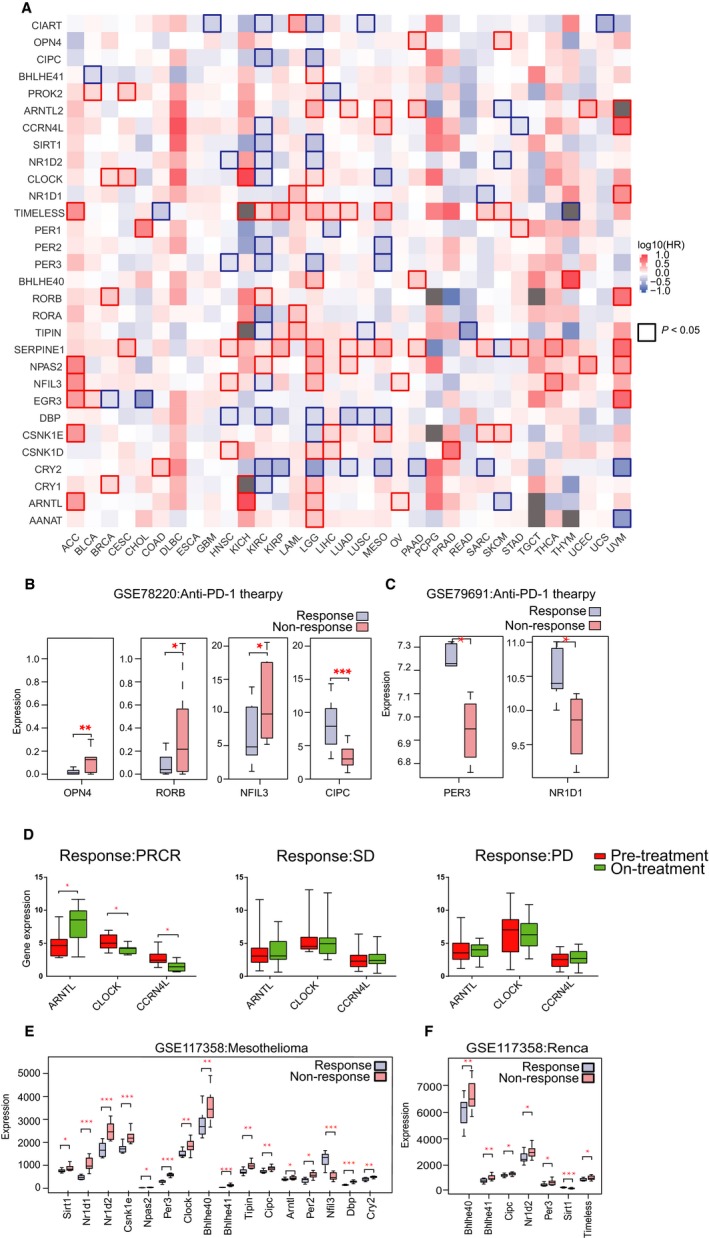
The expression of rhythm genes predicts prognosis and sensitivity of immunological checkpoint blockade therapy. A, The expression of rhythm genes predicts the prognosis of pan‐cancer. Red represents a risk factor with HR value more than 1, whereas blue represents a protective factor with HR <1, and the border‐box indicates statistical significance. B, Comparing the expression of rhythm genes in the Pembrolizumab response group and nonresponse group of melanoma patients. *Indicates *P* < .05, **Indicates *P* < .01, and *** indicates *P* < .001. C, Comparing the expression of rhythm genes in the Nivolumab response group and nonresponse group of RCC patients. *indicates *P* < .05. D, The significantly changed rhythm genes in the melanoma patients responding to anti‐CTLA‐4 treatment. PRCR means partial response or complete response, SD means stable disease, PD means progressive disease. E, Comparing the expression of rhythm genes in the anti‐PD1 and anti‐CTLA4 response group and nonresponse group of mouse mesothelioma samples. *Indicates *P* < .05, **Indicates *P* < .01, and *** indicates *P* < .001. F, Comparing the expression of rhythm genes in the anti‐PD1 and anti‐CTLA4 response group and the nonresponse group of the kidney cancer animal model. *Indicates *P* < .05, ** indicates *P* < .01, and ***Indicates *P* < .001

We demonstrated that rhythm genes are associated with genome instability and are correlated with the infiltration of immune cells in pan‐cancer, which implies that these rhythm genes may play critical roles in the prognosis of cancer patients. We then analyzed the prognosis value of rhythm genes in pan‐cancer and found that the expression of most rhythm genes can predict either positive or negative prognosis of various cancer patients (Figure 6A). Among them, certain rhythm genes such as DBP, CRY2, and CIART are protective effectors for the prognosis of most cancers, whereas certain rhythm genes such as TIMELESS and SERPINE1 are risk factors for the prognosis of most cancer. As expected, certain rhythm genes such as DBP that positively correlated with the infiltration of T lymphocytes also predict benefit prognosis, whereas certain rhythm genes such as SERPINE1, NPAS2, NFIL3, and CSNK1D that negatively correlated with the infiltration of T lymphocytes predicts poor prognosis of cancer patients. Moreover, CART, SIRT1, and PER2 negatively correlated with the infiltration of M2 cells show good prognosis of cancer patients, while SERPINE1 and NFIL3 positively correlated with the infiltration of M2 cells show poor prognosis of cancer patients. Unexpectedly, we also found that certain rhythm genes such as SERPINE1, TIMELESS, NFIL3, and ARNTL2, which are positively correlated with the infiltration of all immune cells, are risk factors for cancer prognosis in pan‐cancer, suggesting that it is necessary to consider the comprehensive effects of cancer progression and cancer immunity caused by genome instability when evaluating the roles of certain rhythm genes in the prognosis of cancer.

### 3.6 The expression of rhythm genes is associated with the sensitivity of immune checkpoint blockade therapy

It has been demonstrated that the infiltration of immune cells in the tumor microenvironment is closely related to checkpoint blockade therapy. Thus, we analyzed the relationship between rhythm gene expression profile and efficacy of anti‐PD1 and anti‐CTLA4. By analyzing GEO data about Pembrolizumab (anti‐PD1 antibody)‐treated melanoma patients, we showed that NFIL3, PORB, and OPN4 were highly expressed whereas CIPC was lowly expressed in the nonresponse group compared with the response group (Figure 6B), which suggests that these rhythm genes may play an opposite function in anti‐PD‐1 therapy, and their expression level may predict responding of melanoma patients to anti‐PD1 therapy. However, in a human renal cell carcinoma dataset with anti‐PD‐1 treatment, we revealed that NFIL3 is highly expressed in the response group compared with the nonresponse group (Figure 6C). The reason why NFIL3 differently expressed in the response of anti‐PD1 therapy in melanoma and renal cell carcinoma may be that NFIL3 expression is associated with infiltration of different immune cells in these two cancer types. In melanoma, the expression of NFIL3 is negatively correlated with the infiltration of CD8^+^ T cells (Figure 5D), thus high expression of NFIL3 predicts nonresponse of anti‐PD1 therapy (Figure 6B), whereas, in renal cell carcinoma, the expression of NFIL3 is positively correlated with the infiltration of CD4^+^ T cell (Figure 5C), and thus predicts response of anti‐PD1 therapy (Figure 6C). In another dataset, we revealed that the expression of ARNTL increased whereas CCRN4L and CLOCK decreased significantly after anti‐CTLA4 treatment in the partial or complete response group, whereas there is no expression change for these potential markers in the stable or progressive group (Figure 6D). In the mesothelioma animal model treated with anti‐PD1 and anti‐CTLA4, we revealed that a lower expression of most rhythm genes predicts response to combined ICB therapy (Figure 6E). Additionally, we also identified that lower expression of several rhythm genes is associated with the sensitivity of combined anti‐PD1 and anti‐CTLA4 therapy in the kidney cancer animal model (Figure 6F). Notably, the lower expression of Bhlhe40, Bhlhe41, Nr1d2, and Cipc predicts sensitivity of combined ICB therapy in both the different models (Figure 6E,F).

## 4 DISCUSSION

In this study, we detected the expression changes of 32 rhythm genes in 33 cancer types and found that most of them were significantly altered partially due to abnormal methylation in various cancer types because their expression levels negatively correlated with the methylation level of these gene promoters. Unexpectedly, we also showed that the expression of several rhythm genes is positively correlated with their methylation level, which is different from the general principle that the expression level of genes is negatively correlated with the methylation level of their promoter. However, the expression level of genes could be positively correlated with the level of methylation within the transcribed regions of genes,^37^ although the mechanisms regarding the intragenic DNA methylation promoting gene expression is still unclear. Since some genes have various transcripts resulting from the alternative promoter or transcription start site, thus it is conceivable that DNA methylation in certain rhythm genes may activate their transcription.

Additionally, we found that certain rhythm genes regulate the expression of other rhythm genes by functioning as a transcription factor in various cancer types. Especially, we found that there are some rhythm genes which regulate the transcription of themselves. For example, NFIL3 is a well‐documented transcriptional repressor,^38^ and there are a number of research works that showed that its expression is elevated in various cancer types and high expression of NFIL3 is associated with malignant hallmarks of cancer^39^, ^40^, ^41^, ^42^ However, in our study, we revealed that this rhythm gene is universally downregulated at the mRNA level in all cancer types compared with their normal tissues, which is different from previous studies. We revealed that NFIL3 could regulate the transcription of itself since it is a transcription repressor, thus it is possible that elevated NFIL3 protein reversely suppresses the transcription and causes the decrease of mRNA of NFIL3 gene in cancer tissues in a negative feedback manner. However, this hypothesis must be investigated with further experiments.

By gene enrichment analysis, we revealed that the major hallmarks of cancer regulated by rhythm genes are genome instability and mutation, which may partially due to that rhythm genes regulate DNA damage response (DDR). Consistence with this finding, it has been reported that the expressional level of certain rhythm gene tends to change when cells undergo DNA damage. For instance, DNA damage increases the Cry1/Cry2 ratio and stimulates the binding of Cry1/2 to Fbxl3.^43^ Moreover, several previous studies demonstrated that certain rhythm genes are involved in the regulation of DDR. For example, it was found that cells with NPAS2‐knockdown showed reduced DNA damage repair capacity compared to normal cells^44^ TIMELESS knockdown accelerated cellular senescence because TIMELESS played an important role in maintaining genome stability, and loss of TIMELESS inhibited DNA damage repair, which triggered the process of senescence.^45^ PER2 mutations resulted in genome instability in liver cancer cells due to impairing P53 response and inhibiting DDR in a mice model^46^ Although there are several rhythm genes have been linked to keeping genome stability, more works are needed to examine the functions of other rhythm genes and explore the molecular mechanisms in regulating DDR and maintaining genome stability.

Previous studies have shown that many immune parameters, such as circulating naïve T‐cells and some proinflammatory cytokines, show systematic fluctuations in human blood. The circadian system and sleep jointly evoke a unique change in leukocyte traffic and induction of proinflammatory cytokines, which shows a circadian peak during nighttime and are further enhanced by sleep.^47^ Other studies also reported the importance of circadian rhythm on the activation of the innate immune system in the onset of inflammatory diseases.^48^, ^49^ Rhythm gene Bmal1 enhances gastric neutrophil recruitment and increases IL‐1α expression and thus promotes gastric contractile changes in a restricted high‐fat diet mice model.^50^ Moreover, loss of the function of Bmal1 exacerbates infiltration of inflammatory cells into inflamed skin in mice model.^51^ Although there are researches revealed that circadian rhythm and certain rhythm genes were associated with the activation of the innate immune system, there is no research that detected the roles of rhythm genes in the infiltration of innate and adaptive immune cells in pan‐cancer. In this study, we investigated the correlation between the expression of rhythm genes and infiltration of immune cells since we found those rhythm genes are associated with genome instability and gene mutation. We revealed that almost all rhythm genes are associated with all immune cell infiltration in pan‐cancer. Specifically, half of them are positively correlated, whereas three of them are negatively correlated with the infiltration level of four detected tumor‐infiltrating immune cells in pan‐cancer. This study implies that rhythm genes play important roles in cancer immunity. Consistence with this finding, we not only found that the expression of rhythm genes associated with prognosis of cancer patients but also revealed that the differential expression of some rhythm genes in patients with metastatic melanoma is closely related to the efficacy of checkpoint blockade therapy. Notably, we found that some of the rhythm genes positively correlated with immune infiltration, which indicates a poor prognosis, whereas certain genes negatively correlated with immune infiltration and shows benefit prognosis. This controversial result about certain genes in cancer immunity and prognosis is also reported previously. For example, PD‐L1 has been found highly expressed in various solid cancer, and associated with high infiltration of cytokine immune cells but usually indicated poor prognosis for patients.^52^

In summary, this study conducted a comprehensive and systematic analysis of the expression and functions of rhythmic genes through public data from 33 cancer types, and showed that the expression of rhythm genes is significantly changed in pan‐cancer, and revealed that rhythm genes are associated with genome instability and mutation and are correlated with the infiltration level of immune cells in cancer microenvironment. This study not only provides a comprehensive understanding of the roles of rhythm genes in pan‐cancer but also may develop a novel method for the diagnosis and treatment of malignant cancer in the future.

## Data Availability

All data used in the article can be obtained in a public database.
